# Investigation of arterial blood flow changes according to compression level in a practical blood flow restriction training method: A cross-sectional experimental study

**DOI:** 10.1371/journal.pone.0359341

**Published:** 2026-10-08

**Authors:** Meltem Ozagir, Sergen Devran, Orkun Akkoc, Bülent Bayraktar, Ilker Yucesir

**Affiliations:** 1 Department of Recreation, Faculty of Sport Sciences, Istanbul Gelisim University, Istanbul, Turkey; 2 Department of Sports Medicine, Istanbul Medical Faculty, Division Of Medical Sciences, Istanbul, Turkey; 3 Department of Movement and Training Sciences in Sports, Faculty of Sports Sciences, Istanbul University-Cerrahpasa, Istanbul, Turkey; 4 Department of Sports Health Sciences, Faculty of Sports Sciences, Istanbul University-Cerrahpasa, Istanbul, Turkey; University of Mississippi, UNITED STATES OF AMERICA

## Abstract

**Background:**

Blood flow restriction (BFR) training is used to partially restrict limb blood flow during exercise. Practical blood flow restriction (pBFR) applications often rely on subjective pressure scales; however, the extent to which perceived compression levels influence arterial blood flow remains unclear. This study aimed to examine changes in popliteal artery blood flow velocity across different subjective compression levels during pBFR.

**Methods:**

This cross-sectional observational study included 42 healthy physically active adults (18–37 years). pBFR was applied to the dominant thigh using a 5 cm elastic band wrapped twice around the proximal thigh. Participants reported perceived compression as free flow (0/10), light (3–4/10), moderate (6–7/10), and high (9–10/10). Popliteal artery blood flow velocity and vessel diameter were measured using Doppler ultrasound. Repeated-measures ANOVA was used to compare outcomes across compression levels. Pearson correlation analyses were performed to examine associations among blood flow velocity, thigh circumference, and skinfold thickness. Analyses were conducted using pooled male and female data.

**Results:**

Blood flow velocity progressively decreased with increasing compression. Significant differences were observed between all compression levels (p < 0.001). Blood flow velocity values were significantly correlated across compression levels. No significant associations were found between anthropometric measures and blood flow velocity. Complete arterial occlusion was not observed in any participant.

**Conclusions:**

Subjective pBFR compression levels were associated with graded reductions in popliteal artery blood flow velocity under the present experimental conditions. These findings reflect acute hemodynamic responses at rest and should not be generalized to exercise adaptations. Anthropometric characteristics were not associated with blood flow responses. Further research is needed to determine the translational relevance of subjective compression levels during exercise.

## Introduction

Blood flow restriction (BFR) is a method used to partially or completely restrict blood flow, or to reduce arterial inflow while occluding venous outflow, through compression applied to the proximal portion of an extremity [1,2]. This method induces local ischemia and hypoxia in the applied region, increases physiological stress, and leads to earlier recruitment of type II muscle fibers during exercise [3]. In addition, the hemodynamic stress induced by BFR has been reported to promote favorable adaptations in the vascular system [4]. Based on these physiological responses, BFR is widely used to induce muscle hypertrophy and to increase muscle strength, particularly during low-load resistance training. The literature emphasizes that BFR training performed at intensities of 20–30% of one-repetition maximum (1RM) is commonly preferred in both clinical applications and sports-related research [5,6]. However, for the purpose of the present study, these training intensity recommendations are only indirectly relevant, as the primary focus was not training adaptation but the hemodynamic response to different compression levels.

In the literature, there are two main compression methods used in BFR applications, depending on the equipment employed. One involves the use of pneumatic cuffs, while the other involves wrapping the limb with elastic bands or elastic straps. In pneumatic cuff systems, pressure is measured using a manometer, and this method is considered the most reliable approach in clinical practice and scientific research because it allows the applied pressure to be determined according to the individual and the cuff size used. However, the use of pneumatic cuffs in field settings and with large groups of athletes is less feasible and less practical [7,8].

To overcome these limitations, Loenneke and Pujol introduced the use of elastic bands or semi-elastic fabric straps instead of pneumatic cuffs [7]. This approach, has been defined as practical blood flow restriction (pBFR), in which BFR is applied using elastic bands or straps rather than pneumatic cuff systems [7].

The low cost of pBFR, its rapid application, and its feasibility in training practices involving large groups provide significant practical advantages. Studies in the literature have reported that pBFR produces outcomes similar to those of pneumatic cuff systems, including muscle hypertrophy and strength gains [9–13], improvements in sprint performance [11], and increases in maximal oxygen uptake [14]. Although elastic systems may offer practical advantages during exercise, this statement should be interpreted cautiously, as direct biomechanical comparisons with pneumatic systems remain limited [7]. The cuff pressure or tightness level of the elastic band is an important factor in achieving the targeted physiological adaptations underlying the BFR mechanism [8]. In the pBFR method, standardizing the wrapping pressure of the elastic band is challenging; however, it is possible to subjectively define the perceived tightness or compression. Wilson et al. adopted a rating method based on perceived compression during pBFR application using elastic bands [15]. According to this method, the pressure scale ranges from 0 to 10, where zero (0) represents free flow condition and ten (10) represents the highest perceived compression level. In their study, a compression level of 7 out of 10 was identified as the highest level at which participants were able to exercise without complete arterial occlusion, and resistance training was performed at this tightness level. At a compression level of 6–7, arterial blood flow was maintained, whereas venous occlusion occurred based on ultrasound measurements [15,16].

In a study conducted by Bell et al. [16], individuals undergoing pBFR were able to identify pressures below occlusion pressure based on their perceived tightness level (7/10). Importantly, Bell et al. [16] used a pneumatic cuff to apply the BFR pressure, in contrast to the elastic band used in the present study and the practical BFR approach used by Wilson et al. [15]. However, it is important to note that the difference between arterial occlusion pressure and the 10/10 perceived tightness condition was not statistically significant (difference = 7 mmHg [−3, 18]), indicating overlap between subjective perception and actual occlusion pressure. Furthermore, a follow-up study by the same research group demonstrated limited test–retest reliability of the perceived tightness scale across multiple testing sessions, with substantial within-subject variability in arterial occlusion pressure despite similar perceptual ratings. These findings that perceptual scaling methods may have important limitations for standardizing pBFR pressure across repeated sessions and suggest reduced confidence in the use of a fixed 7/10 threshold [17].

Despite the observed benefits of BFR methods applied during exercise, evidence regarding changes in blood flow at different perception-based pressure levels remains limited. Moreover, limited and inconsistent data are available in the literature regarding the standardization of applied pressure and the magnitude of blood flow restriction achieved with pBFR methods. The term “inconsistent” refers particularly to variability across studies in reported relationships between cuff tightness, arterial occlusion pressure, and actual blood flow restriction outcomes, with some studies reporting strong associations while others report weak or non-significant relationships [8,18]. Therefore, determining the extent to which arterial blood flow is restricted at different compression levels using the pBFR method is of considerable importance for both basic and clinical sciences, as well as for exercise science applications. In addition, studies examining the effects of skinfold thickness and limb circumference at the site of restriction on pressure and blood flow are limited and report inconsistent findings, with some studies suggesting positive associations between limb size and required occlusion pressure, while others report weak or non-significant relationships [8,17].

Within this framework, the primary aim of the present study was to examine changes in blood flow in the popliteal artery at different compression levels free flow (0), light (3–4), moderate (6–7), and high (9–10) using a subjective pressure perception scale. The secondary aim was to determine the relationship between the perceived restriction levels and blood flow velocity measured in the popliteal artery. It was hypothesized that increasing perceived compression would result in significant differences in popliteal arterial blood flow and that perceived compression levels would be associated with blood flow velocity. In addition, it was hypothesized that participants with greater skinfold thickness and larger thigh circumference would exhibit a smaller reduction in blood flow in response to compression [8,18].

## Materials and methods

The study protocol was conducted in accordance with the Declaration of Helsinki and was approved by the İstanbul University–Cerrahpaşa Non-Interventional Clinical Research Ethics Committee (Approval No. 2023/104). All participants provided written informed consent prior to participation. The data were collected at the Sports Medicine Laboratory of Istanbul University in 2023. The present study was not prospectively registered or pre-registered.

### Subjects

A total of 42 physically active volunteers (22 men and 20 women) aged between 18 and 37 years (22.37 ± 3.14) participated in the study. Participants were instructed to refrain from caffeine consumption for 8 hours, alcohol consumption for 24 hours, and vigorous physical activity for 24 hours prior to testing and measurements. Exclusion criteria included pregnancy, blood coagulation disorders, cardiovascular diseases, a history of lower-extremity surgery within the previous 12 months, or the use of medications affecting blood flow. Participants were recruited between October 2023 and December 2023.

### Study design

This was a cross-sectional observational study with a repeated-measures design. The study was conducted in two separate sessions with an interval of three days between sessions. Throughout the study period, participants were instructed to avoid any strenuous physical activity. The first session was a familiarization session. During this session, participants were informed about the purpose and procedures of the study, screened for eligibility, provided written informed consent, and were introduced to the compression band. Participants were allowed to familiarize themselves with the material through self-application. During this session, participants applied the elastic band to their legs for a few minutes by wrapping it twice in the manner to be used in the study, with assistance from the researcher, at compression levels they personally selected. This process allowed participants to become familiar with the sensation of compression produced by the elastic band and with rating the perceived compression level. During the second visit, height, body mass, and thigh measurements were obtained prior to ultrasound assessments. Thigh circumference of the dominant leg to which pBFR would be applied was measured using a measuring tape, and skinfold thickness was measured using a caliper. All measurements were performed in a quiet, temperature-controlled room (22°C). A commercially available pBFR elastic band, 90 cm in length and 5 cm in width, was used in the study. The band was applied in two adjacent wraps around the proximal portion of the dominant thigh, resulting in an effective application width of approximately 10 cm. However, this configuration should not be interpreted as mechanically equivalent to a true 10 cm pneumatic cuff, as elastic and pneumatic systems may differ in pressure distribution characteristics. Blood flow velocity under free-flow conditions (0/10) was measured first and considered the baseline measurement prior to compression. Light (3–4/10), moderate (6–7/10), and high (9–10/10) compression levels were then applied in randomized order. A 10-minute interval was provided between conditions to reduce carryover effects and allow partial return toward baseline, consistent with previously used acute BFR protocols. However, complete recovery between conditions cannot be fully guaranteed.

Thigh circumference and skinfold thickness measurements were obtained prior to blood flow assessments. All anthropometric measurements were performed at the midpoint of the thigh of the dominant leg, corresponding to the same anatomical region where the pBFR cuff was applied. Each measurement was taken in duplicate, and the mean value was used for statistical analyses to improve measurement reliability and reduce potential measurement error. Measurements were performed by the same experienced investigator under standardized laboratory conditions.

### Height and weight measurement

Participants’ height was measured using a SECA 202 stadiometer (SECA GmbH, Hamburg, Germany) with an accuracy of ±1 cm, while standing upright with feet together and the head positioned in the Frankfurt plane. Body mass was measured using an electronic scale (SECA GmbH, Hamburg, Germany) with a precision of 0.1 kg.

### Thigh circumference measurement

Thigh circumference was measured using a Gulick anthropometric tape (Lafayette Instrument Company, Lafayette, IN, USA) at the midpoint of the thigh while the participant was standing upright with feet approximately 10 cm apart and body weight evenly distributed between both legs [19,20].

### Skinfold measurement

Skinfold thickness was measured vertically at the midpoint between the inguinal crease and the proximal border of the patella while the participant was standing with the leg relaxed [21,22]. The skin and subcutaneous fat tissue were grasped gently between the thumb and index finger and pulled slightly away from the underlying muscle tissue in the natural direction of the skinfold. The jaws of the caliper were applied perpendicular to the fold, and the skinfold was compressed using the constant pressure of the caliper. The thickness of the skin and subcutaneous fat tissue was then read in millimeters from the gauge [23]. All skinfold measurements were performed by the same experienced investigator to remove inter-rater variability. Each measurement was taken three times, and the mean value was used for statistical analysis.

### Elastic band blood flow restriction method

The pBFR elastic band used in the study (Strade Store) was a commercially available product. The elastic band was applied horizontally to the proximal portion of the participants’ dominant thighs, starting medially at the level of the inguinal crease and including the adductor muscles. The band was wrapped in two adjacent turns without overlap or gaps to achieve a width of 10 cm. Different pressure levels were applied by adjusting the length and tension of the band using the lockable buckle located at the end of the band (Fig 1).

**Fig 1:**
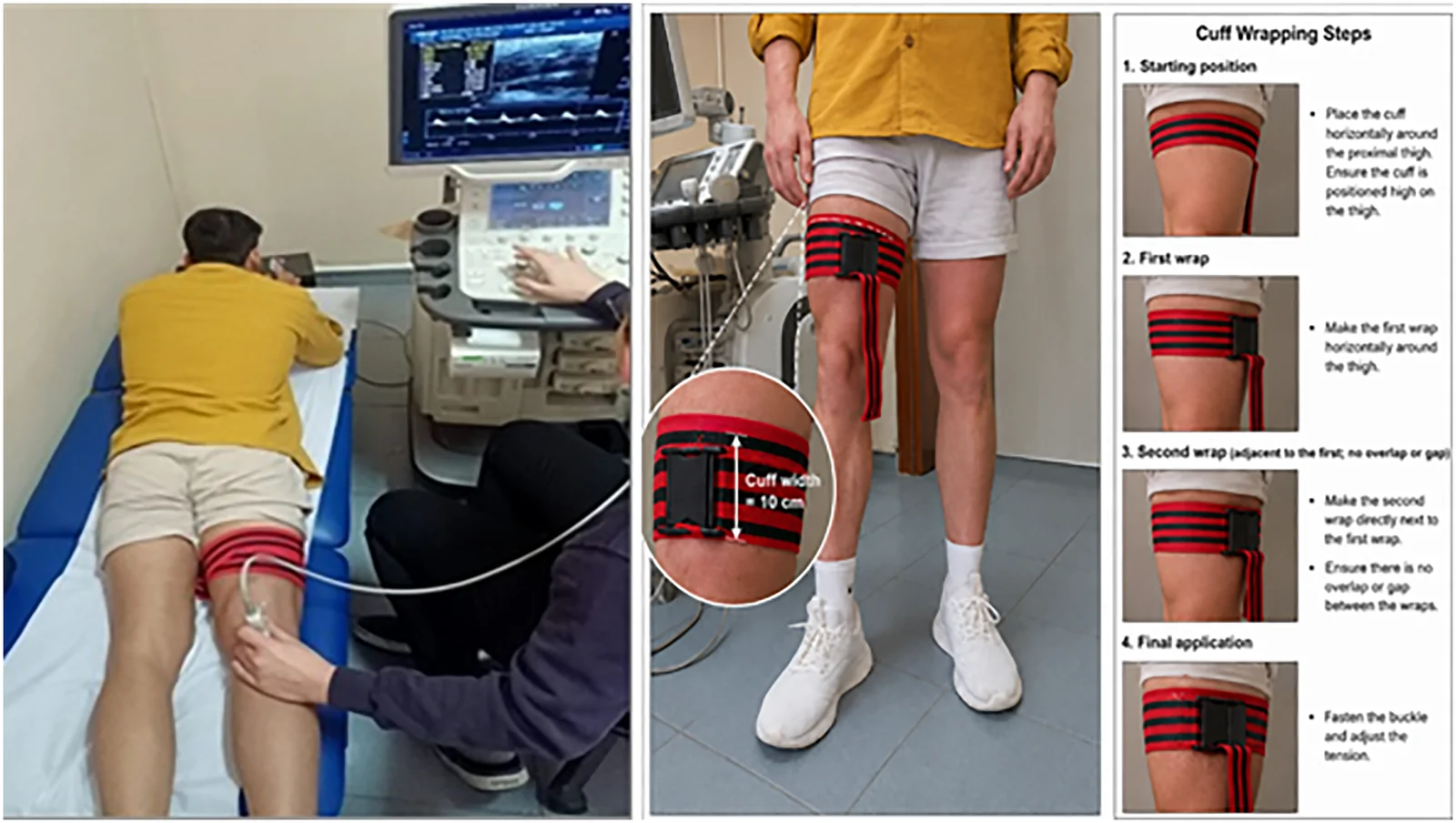
Application of pBFR and measurement of blood flow velocity using Doppler ultrasound.

Prior to measurements, the compression band was wrapped around the application site for familiarization purposes and tightened to compression levels representative of the experimental conditions (light 3–4/10, moderate 6–7/10 and high 9–10/10). Participants were asked how much pressure they perceived in order to ensure that they clearly understood and could describe the sensation of compression, thereby reinforcing the familiarization process beyond the initial session. Blood flow velocity was first measured under free-flow conditions (0/10). Participants were then sequentially exposed to light (3–4/10), moderate (6–7/10), and high (9–10/10) perceived compression levels. This progressive sequence allowed participants to compare each compression level with the previous one and facilitated accurate perception of the intended level of tightness. Immediately after the target compression level was achieved, Doppler ultrasound measurements were initiated. A 10-minute interval was allotted between different compression conditions.

### Assessment of perceived pressure (Visual Analog Scale, VAS)

In this study, a visual analog scale (VAS) was used to determine the pressure perceived during the application of the pBFR elastic band. The VAS allows values that cannot be measured numerically to be expressed in numerical form [24]. The perceived pressure/tightness scale was introduced to the participants after being adapted from Wilson et al. [15]. Due to participants’ difficulty in defining precise numerical values, three consecutive value ranges were determined in the study, and participants were asked to report the pressure they perceived within these ranges. Accordingly, the conditions were defined as follows: free flow condition (0), light compression (3–4), moderate compression (6–7), and high compression (9–10).

### Ultrasound measurement

The popliteal artery was selected as the measurement site due to its suitability for Doppler ultrasound assessment of lower-limb blood flow in a standardized supine position. From a methodological perspective, peripheral arterial sites such as the popliteal artery have demonstrated acceptable intra- and inter-rater reliability in Doppler-based blood flow velocity assessments [25] and allow relatively stable probe positioning during repeated measurements. Compared with more proximal vessels such as the femoral artery, the popliteal artery may provide more consistent ultrasound acquisition due to its superficial location in the posterior knee region [25,26]. From a physiological perspective, the popliteal artery supplies the distal musculature of the lower limb, which is the primary region affected by proximal blood flow restriction, making it suitable for assessing peripheral hemodynamic responses during pBFR applications [27].

To minimize measurement bias, all ultrasound assessments were performed by a single experienced investigator under standardized environmental conditions. Participants were positioned prone, and the Doppler probe was placed over the posterior aspect of the knee to visualize the popliteal artery (Fig 1). A Toshiba Aplio 500 ultrasound system (Toshiba Medical Systems Corporation, Otawara, Japan) was used. Mean blood flow velocity (V_mean) and vessel diameter (V_d) were assessed using a linear probe (10 MHz imaging frequency; 5 MHz Doppler frequency). Doppler spectra and images were recorded before and during blood flow restriction. Vessel diameter was calculated as the mean of three measurements obtained across three non-consecutive cardiac cycles during the final 15 seconds of each condition. Mean blood flow velocity was obtained with an insonation angle maintained at ≤60°. Blood flow velocity values were recorded at 10-second intervals and averaged. For each compression level, Doppler measurements were obtained twice within the same application period, and the mean values were used for analysis. The 10-minute interval was applied only between different compression conditions, not between repeated measurements.

Blood flow volume (V_flow) was calculated using the formula:


$$\begin{array}{c} \text{V}\_\text{flow}=\text{V}\_\text{mean}\times \pi \times {\left(\text{V}\_\text{d}/\text{2}\right)}^{\text{2}}\times \text{60} \end{array}$$

Each condition was measured twice, and mean values were used for analysis.

### Statistical analyses

All statistical analyses were performed using JASP statistical software (Version 0.95; JASP Team, Amsterdam, The Netherlands). Descriptive statistics are presented as mean and standard deviation (SD) for all demographic variables. The normality of the data was assessed using skewness and kurtosis values. Values between −1.5 and +1.5 were considered indicative of a normal distribution, and the data were found to be normally distributed. Measurements obtained at different time points were analyzed using repeated-measures analysis of variance (ANOVA). Pairwise comparisons between time points were conducted using estimated marginal means with Fisher’s Least Significant Difference (LSD) adjustment. To assess the robustness of the findings, a sensitivity analysis using the Holm–Bonferroni correction was also performed. Proportional reductions were calculated at the individual participant level using each participant’s free-flow value as baseline. Individual percentage changes were then computed for each compression level and averaged across participants to obtain group-level estimates. The proportional reduction was calculated using the following formula: % reduction = [(free-flow velocity − velocity at compression level)/ free-flow velocity] × 100.

Pearson product-moment correlation coefficients (r) were calculated to evaluate the relationships between compression level, thigh circumference, skinfold thickness, and blood flow. The magnitude of associations was classified according to absolute r values as low (|r| < 0.30), moderate (0.30 ≤ |r| < 0.50), and high (|r| ≥ 0.50).

Pearson correlation analyses were performed using pooled observations across compression conditions; therefore, repeated measurements within participants were treated as independent observations for the purpose of correlation analysis. This limitation is acknowledged in the interpretation of the results. Prior to correlation analyses, multicollinearity among anthropometric variables was assessed using correlation matrix inspection (and/or variance inflation factor where applicable), and no evidence of problematic multicollinearity was observed. The assumption of sphericity was tested using Mauchly’s test, which indicated a significant violation (W = 0.174, χ²(5) = 69.385, p < .001). Therefore, Greenhouse–Geisser correction was applied (ε = 0.485) to adjust the degrees of freedom for all within-subject effects and to control for potential inflation of Type I error. Post-hoc pairwise comparisons were conducted using estimated marginal means, and results were interpreted with reference to adjusted significance levels.

A sensitivity analysis was conducted using G*Power (version 3.1.9.7; Heinrich Heine University Düsseldorf, Germany) to determine the minimum detectable effect size given the achieved sample size. The analysis was based on a repeated-measures within-subject design including 42 participants and four measurement time points. The model assumed an alpha level of 0.05, a correlation among repeated measures of 0.50, and a Greenhouse–Geisser sphericity correction (ε = 0.48). The analysis indicated that the study was capable of detecting a minimum effect size of f = 0.303 under the specified assumptions.

## Results

Participant characteristics are presented in Table 1. The mean age of the participants was 22.37 ± 3.14 years, the mean height was 172.00 ± 8.42 cm, the mean body weight was 66.97 ± 12.14 kg, and the mean body mass index (BMI) was 22.40 ± 2.86 kg/m². Mean thigh circumference was 53.02 ± 8.16 cm, and mean thigh skinfold thickness was 24.29 ± 6.70 mm.

**Table 1:** Participants characteristics.

Variable N = 42	Total (min. - max.)	Total (mean ± SD)
Age (years)	18–37	22.37 ± 3.14
Height (cm)	155–194	172 ± 8.42
Body Weight (kg)	45–103	66.97 ± 12.14
Body Mass Index (BMI)	17.24–32.51	22.40 ± 2.86
Thigh Circumference (cm)	40–65	53.02 ± 8.16
Thigh Skinfold Thickness (mm)	15–40	24.29 ± 6.70

max: maximum; min: minimum; Std. Dev.: standard deviation; N: number of participants.

Repeated measures ANOVA revealed a statistically significant difference between measurements, *F* = 369.75, *p* < .001, ηp² = .900. Values are presented as mean ± standard deviation. Bonferroni-corrected pairwise comparisons showed that all measurements differed significantly from each other, as indicated by different superscript letters. Free flow had the highest value, followed by light, moderate and high pressure, respectively, demonstrating a progressive decrease across measurements. The overall ranking was free flow > light > moderate > high pressure. ηp² = partial eta squared (Table 2).

**Table 2:** Blood flow values across compression levels and repeated-measures ANOVA results.

Free Flow	Light	Moderate	High Pressure	F	p	ηp²
58.55 ± 15.71^a^	44.98 ± 9.43^b^	32.79 ± 7.43^c^	17.21 ± 6.01^d^	369.75	<.001	.900

F = F statistic; p = significance value; ηp² = partial eta squared.

Mean differences, standard errors, 95% confidence intervals, and Bonferroni-adjusted *p*-values are presented for pairwise comparisons between repeated measurements. Positive mean differences indicate that the first measurement in the comparison had a higher value than the second measurement. Accordingly, free flow was significantly higher than light, moderate, and high pressure; light was significantly higher than moderate and high pressure; and moderate was significantly higher than high pressure. Conversely, high pressure had significantly lower values than free flow, light, and moderate; moderate was significantly lower than free flow and light; and light was significantly lower than free flow. All pairwise comparisons were statistically significant after Bonferroni correction (*p* < 0.001), indicating an overall ranking of free flow > light > moderate > high pressure (Table 3).

**Table 3:** Pairwise comparisons of repeated measurements with LSD adjustment.

Comparison	Mean Difference	Standard Error	95% Confidence Interval Lower Bound	95% Confidence Interval Upper Bound	p	Interpretation
**1–2**	13.571	1.400	9.691	17.452	<0.001	1 > 2
**1–3**	25.762	1.720	20.995	30.529	<0.001	1 > 3
**1–4**	41.333	2.245	35.110	47.557	<0.001	1 > 4
**2–3**	12.190	0.777	10.035	14.345	<0.001	2 > 3
**2–4**	27.762	1.195	24.449	31.075	<0.001	2 > 4
**3–4**	15.571	1.012	12.767	18.376	<0.001	3 > 4

p = significance value.

Significant positive correlations were observed between blood flow velocities across all compression levels (r = 0.377–0.855, all p < 0.05). Thigh circumference was positively correlated with skinfold thickness (r = 0.391, p = 0.010). Furthermore, baseline blood flow velocity was positively associated with skinfold thickness (r = 0.330, p = 0.033); however, no significant relationships were observed between skinfold thickness and blood flow velocity at any compression level (all p > 0.05) (Table 4).

**Table 4:** Correlation analysis of thigh circumference, skinfold thickness and blood flow velocity.

Variable		Free flow (0)	High (9–10)	Moderate (6–7)	Light (3–4)	Leg Circumference (cm)	Skin Thickness (mm)
**Free flow (0)**	**Pear. K.** **P**	1	0.377 0.014	0.761 0.000	0.855 0.000	0.219 0.164	0.330 0.033
**High (9/10)**	**Pear. K.** **P**	0.377 0.014	1	0.541 0.000	0.574 0.000	−0.065 0.684	0.114 0.473
**Moderate (6/7)**	**Pear. K.** **P**	0.761 0.000	0.541 0.000	1	0.847 0.000	0.104 0.510	0.213 0.177
**Light (3/4**	**Pear. K.** **P**	0.855 0.000	0.574 0.000	0.847 0.000	1	0.153 0.332	0.199 0.207
**Leg Circumference (cm)**	**Pear. K** **P**	0.219 0.164	−0.065 0.684	0.104 0.510	0.153 0.332	1	0.391 0.010
**Skin Thickness (mm)**	**Pear. K** **P**	0.330 0.033	0.114 0.473	0.213 0.177	0.199 0.207	0.391 0.010	1

Pear. K = Pearson correlation coefficient; P = significance value; cm = centimeters; mm = millimeters p ≤ 0.05.

Mean blood flow was highest during free flow (58.55 mL/min) and progressively decreased at light (44.98 mL/min), moderate (32.79 mL/min), and high pressure (17.21 mL/min). Despite some inter-individual variability, the overall trend was a consistent decline with increasing compression (Fig 2).

**Fig 2:**
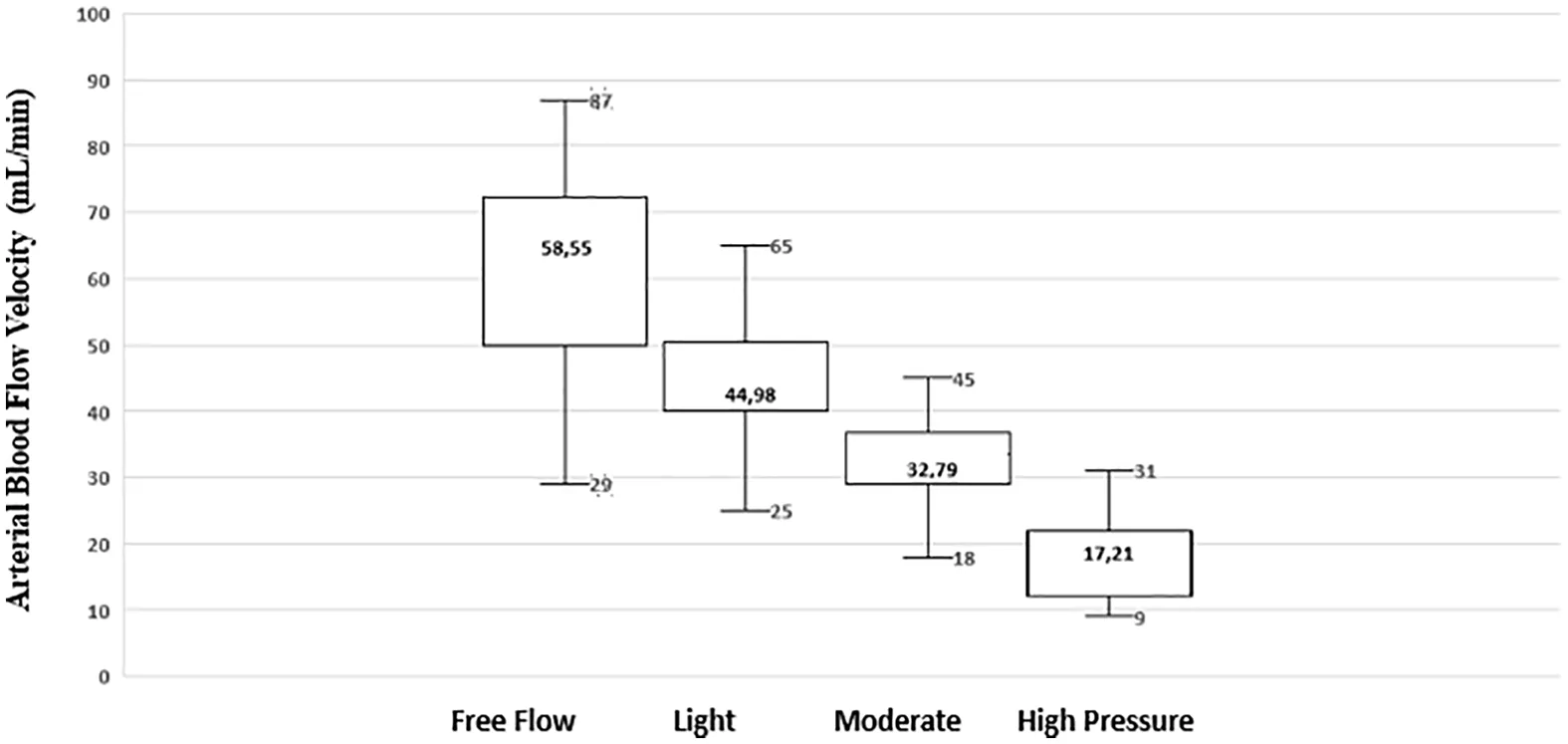
Changes in popliteal artery blood flow across compression levels.

The change ratio of blood flow group averages relative to free flow averages at different restriction levels is shown. Accordingly, the flow ratios at the three levels, from mild to severe, are 76.8%, 56% and 29.3%, respectively (Fig 3).

**Fig 3:**
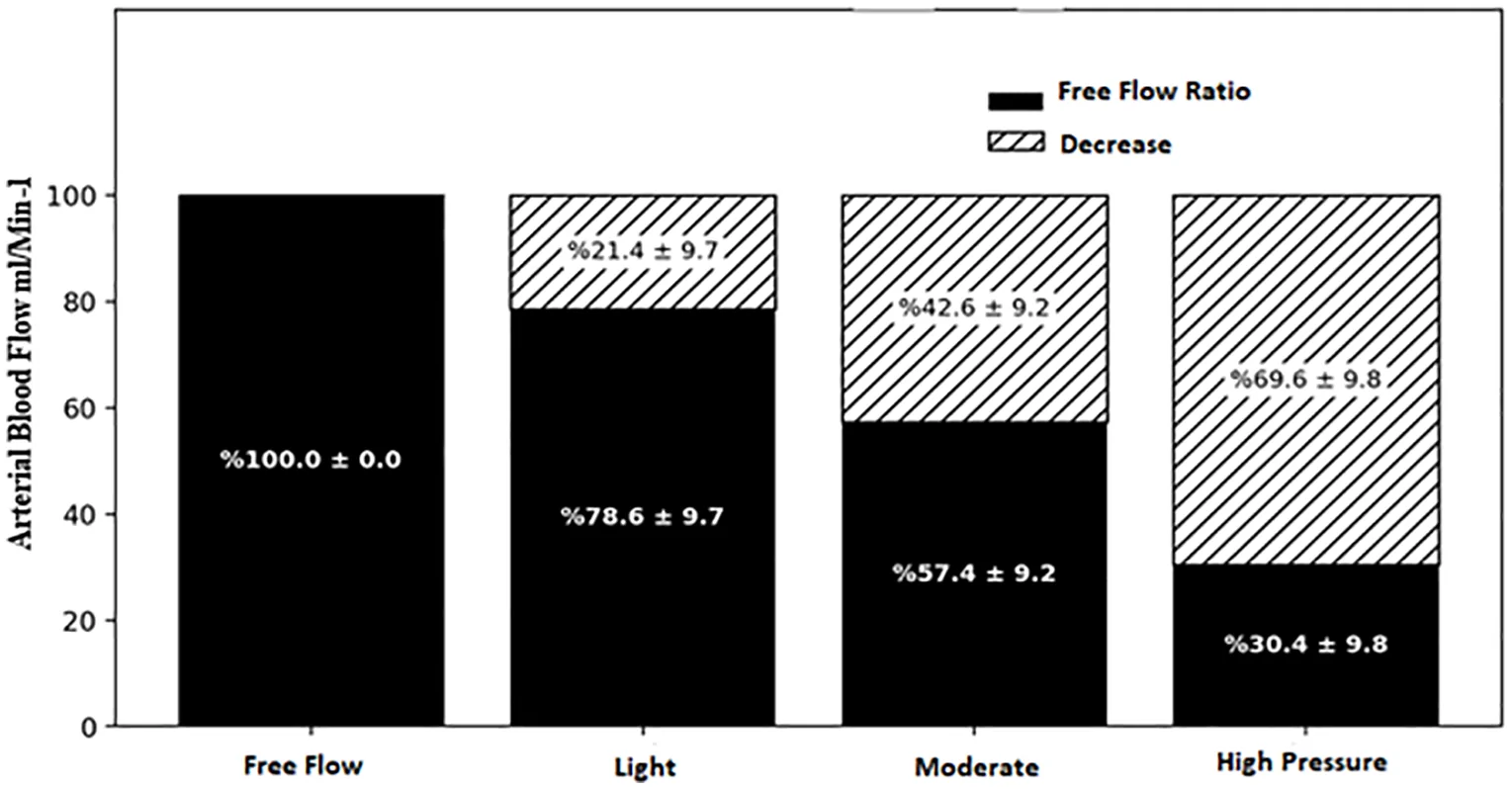
Change in blood flow velocity at different levels of restriction (relative to free flow).

Changes in arterial blood flow across compression levels. Individual responses are shown with thin gray lines, while the blue line represents the group mean. Blood flow gradually decreased from free flow to high pressure, and this downward pattern was fairly consistent among participants. Although there was some variability between individuals, the overall trend was a clear reduction in flow as compression increased (Fig 4).

**Fig 4:**
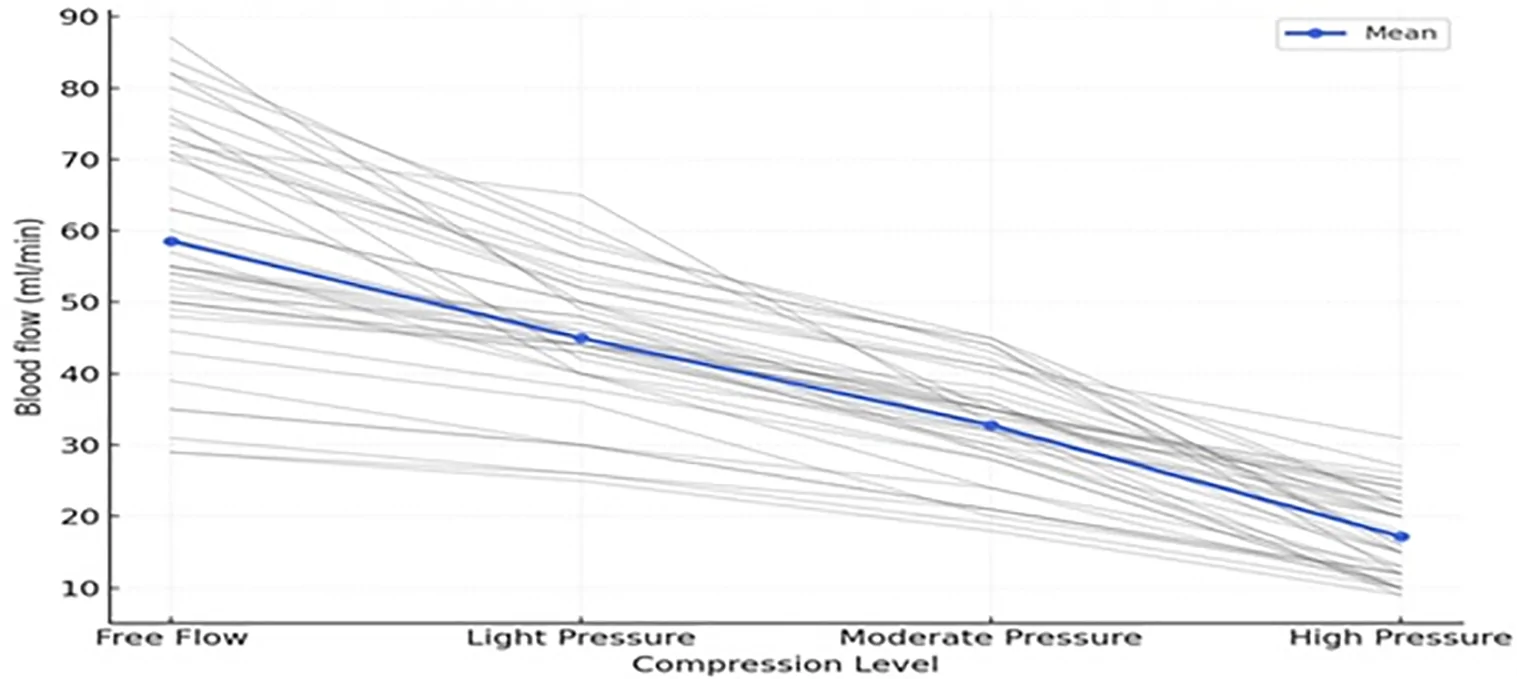
Blood flow velocity of participant at different compression levels.

## Discussion

This study was designed to examine changes in blood flow in the popliteal artery, a major artery of the lower extremity, under different pressure conditions within a framework in which participants reported the level of compression perceived during the application of an elastic blood flow restriction (pBFR) band on a scale from 0 to 10. It was hypothesized that different subjective pressure levels would produce significant differences in blood flow within the popliteal artery and that a significant relationship would be observed between perceived compression (restriction) levels and measured blood flow velocity. In addition, it was hypothetically considered that blood flow values varying according to compression level might provide guidance for obtaining proportional constants, and findings related to this assumption were investigated. It was observed that participants experienced difficulty in expressing perceived compression (pressure) sensations separately for each step of the 10-point scale. Therefore, participants were allowed to report compression levels in ranges representing two adjacent points, and the levels were defined accordingly. In this manner, the first two levels (1 and 2) were omitted, and the following levels were established: 3–4/10 (light), omitting level 5 and defining 6–7/10 (moderate), and omitting level 8 and defining 9–10/10 (high pressure).

The results demonstrated that, with the pBFR method applied to the proximal thigh, mean blood flow velocity was significantly reduced at all compression levels compared with free-flow conditions and that different compression levels had significantly different effects on blood flow velocity. As expected, blood flow velocity decreased as compression level increased (blood flow: free > light > moderate > high pressure). In addition, supporting the concept of determining proportional constants for compression levels, individual proportional changes and group mean proportional changes were very close to each other at all compression levels, being 21.4% for light, 42.6% for moderate, and 69.6% for high pressure compression.

Although the study included 22 male and 20 female participants, sex differences were not included in the statistical analysis due to the limited sample size. However, previous studies have reported that sex does not significantly influence blood flow responses. Bell et al. reported that the effects on blood flow at the same compression level did not differ between female and male participants [16]. Although the decrease in blood flow with increasing compression is an expected outcome, the key finding of the present study is that blood flow velocity decreased significantly between all compression levels despite relatively small subjective differences between adjacent levels. This finding indicates the sensitivity of subjective grading and was evaluated as evidence supporting the reliability of pBFR applications in field settings. In the study conducted by Wilson et al. venous and arterial blood flow levels were examined at different pBFR compression levels applied to the thigh [15]. Their results demonstrated, via ultrasound, that venous flow ceased at level 7 (moderate, pain-free pressure) on a 0–10 tension scale, while both venous and arterial flow ceased at level 10. Similarly, Bell et al. reported that blood flow was present at level 7 in both upper and lower extremities, that this level was pain-free, and that blood flow ceased at level 10 (complete occlusion) [16]. They further suggested that subjective grading using elastic bands may be more practical than pneumatic cuffs, which are more difficult to apply. Wilson et al. likewise reported complete cessation of venous and arterial flow at compression level 10 [15]. It is important to note that both the present study and the study by Wilson et al. [15] used elastic bands, whereas Bell et al. [16] used a pneumatic cuff to apply BFR.

Based on perceived pressure (tightness) scales, a rating of 7 out of 10 (pain-free moderate pressure) has been used as a reference value in previous studies [28,29]. In the present study, the marked reduction in blood flow velocity (42.6%) observed at compression level 6–7 is consistent with the literature; however, unlike previous studies, complete arterial occlusion was not observed in any participant at the severe (9–10/10) level (individual proportional flow reduction: 69.6%, with a residual flow ratio of 30.4%). This observation differs from previously reported findings in the literature. Possible explanations include differences in elastic band materials, anthropometric characteristics of the study population, and the wrapping methodology used. Importantly, this observation is likely related to methodological factors rather than indicating a general limitation of pBFR systems. The absence of complete arterial occlusion in this study may be more plausibly explained by methodological factors, including the characteristics of the elastic band, the wrapping technique, and the resulting pressure distribution, rather than the inherent capacity of pBFR systems. Therefore, caution is warranted when generalizing these results to all practical BFR applications.

Furthermore, recent evidence has highlighted important limitations regarding the reliability of perceived tightness-based scaling methods. Bell et al. demonstrated substantial within-subject variability in arterial occlusion pressure across repeated testing sessions and across multiple days despite similar subjective tightness ratings. These findings suggest that perceptual scaling methods may not provide stable or reproducible estimates across time, which may limit their utility for standardizing pBFR applications in both research and practical settings.

Another finding of the study was that skinfold thickness was related to thigh circumference and that free-flow velocity was related to skinfold thickness. The relationship between skinfold thickness and thigh circumference is expected and natural; however, as these variables are unrelated to compression levels, they represent secondary findings. No relationship was found between thigh circumference or skinfold thickness and blood flow velocity at different compression levels [17]. In the literature, several studies have reported that arm and thigh circumference are determinants of arterial occlusion pressure [9,15]. In a study conducted by Meek et al. involving 42 participants’ significant relationships were observed between limb circumference, skinfold thickness, and BFR applied to the arm, whereas no significant relationships were found when BFR was applied to the leg [18]. The authors attributed this to differences in anthropometric characteristics (muscle and fat composition) of the limb to which BFR was applied.

In the present study, the lack of an effect of thigh circumference and skinfold thickness on blood flow under compression may be explained by the homogeneous structure of the study group and the fact that participants body mass index (BMI) values were within the normal range (group: 22.40 ± 2.86 kg/m²; women: 21.05 ± 2.53 kg/m²; men: 23.63 ± 2.62 kg/m²). However, BMI represents a global measure of body composition and may not adequately capture localized tissue characteristics that influence vascular compression. Therefore, regional anthropometric variables such as thigh circumference and skinfold thickness may be more relevant determinants of pBFR-induced hemodynamic responses. Hunt et al. similarly reported that limb circumference is influential in achieving complete occlusion but is not a determining factor for partial restriction [30]. All studies in the literature examining the effects of limb circumference and skinfold or adipose tissue thickness on restricted blood flow have been conducted using pneumatic cuffs. In the present study, the absence of an effect of leg circumference and skinfold thickness on blood flow across different perceived pressure levels was evaluated as an important finding supporting the widespread field applicability of pBFR and subjective grading, particularly in individuals with BMI values within the normal range.

This study has several limitations. The study was not specifically powered to detect sex-related differences; therefore, analyses were conducted using pooled data from male and female participants. Another potential limitation of the study is that the inability to achieve complete occlusion may have been influenced by the specific cuff characteristics and wrapping methodology employed. Therefore, the findings should not be interpreted as evidence that all practical blood flow restriction approaches are incapable of producing complete arterial occlusion.

In addition, arterial blood flow was assessed under resting conditions, and therefore the findings may not fully reflect hemodynamic responses during exercise, where muscle contractions and metabolic demands could alter blood flow patterns. Furthermore, although the elastic band was wrapped twice to obtain an application width of approximately 10 cm, the mechanical properties and pressure distribution characteristics may differ from those of a true 10 cm pneumatic cuff. Therefore, direct comparisons between elastic pBFR systems and pneumatic cuff systems should be interpreted cautiously. Finally, only one type of elastic band and application technique was used, and different materials or widths may produce different vascular responses. Systemic factors such as systolic blood pressure were not measured in the present study and may also contribute to inter-individual variability in blood flow responses. Therefore, future studies should consider including cardiovascular parameters to better explain the variability observed in pBFR-induced hemodynamic responses.

Occlusion of venous return over time leads to swelling of the limb subjected to BFR, increased tissue stasis, and a reduction in arterial flow. As a result, participants may experience increasing pain and pressure sensations as application duration increases, making prolonged exercise or research protocols particularly challenging. For this reason, the commonly preferred 7/10 compression level in pBFR studies represents an important reference point. In the present study, arterial blood flow was reduced by more than 20% even at the lowest compression level (light 3–4/10), demonstrating that blood flow is reduced at compression levels below the widely accepted 7/10 standard. These findings suggest that subjective compression levels below 7/10 may also warrant consideration in pBFR applications. Future studies should investigate the effects of these lower compression levels on training adaptations and field-based applications. In addition, future research should determine whether subjective perceived compression levels correspond to specific ranges of relative arterial occlusion pressure (%AOP) and evaluate the consistency of these relationships both within individuals across repeated assessments and between individuals. Such findings may contribute to improved standardization and practical implementation of pBFR protocols.

## Conclusions

The present findings indicate that subjective pBFR application involving the wrapping of a commercially available 5-cm-wide elastic band twice around the proximal thigh near the inguinal crease, resulting in an effective width of approximately 10 cm, was associated with a progressive reduction in popliteal artery blood flow under the specific experimental conditions tested. The results demonstrate that pressure applications at closely spaced but distinct levels produce statistically significant differences in blood flow restriction within the limb. The observation that blood flow is restricted to significantly different extents at high (9–10/10), moderate (6–7/10), and light (3–4/10) pressure levels indicates that subjective pBFR applications were associated with graded reductions in blood flow velocity under the conditions of the present study. In this study, leg circumference and skinfold thickness were not associated with blood flow velocity at different compression levels. An important consideration is that the study group consisted of individuals with normal BMI values. Therefore, these findings should be interpreted within the context of a relatively homogeneous sample and may not be generalizable to populations with different anthropometric characteristics.

Complete occlusion was not achieved in any participant, even at a severe subjective intensity of 9–10/10, and arterial blood flow was not fully interrupted. This observation may reflect methodological factors specific to the present protocol, including the characteristics of the elastic band, wrapping technique, and pressure distribution, and therefore should not be generalized to all practical BFR applications. Future studies investigating complete occlusion with elastic bands may benefit from monitoring blood flow using Doppler ultrasound or other objective methods. The present findings are limited to acute hemodynamic responses measured under resting conditions and do not provide information regarding training adaptations, long-term outcomes, or blood flow responses during exercise. Nevertheless, because individual differences in blood flow velocity and volume decreased as compression level increased, higher compression levels were associated with more consistent blood flow responses across individuals under the conditions of the present study. Future studies should focus on how blood flow responds during exercise performed under different compression levels. Within this context, the present study does not provide evidence regarding whether the reductions in blood flow observed at the low compression level are sufficient to induce meaningful physiological or training adaptations. Future studies should investigate this question during exercise conditions, as such information would be valuable for both performance-oriented field applications and research-focused laboratory investigations.

## Supporting information

S1 Data Minimal dataset containing participant characteristics and arterial blood flow measurements at different compression levels. (XLSX)
